# Meta-Analysis: Diagnostic Accuracy of Anti-Carbamylated Protein Antibody for Rheumatoid Arthritis

**DOI:** 10.1371/journal.pone.0159000

**Published:** 2016-07-20

**Authors:** Liubing Li, Chuiwen Deng, Si Chen, Shulan Zhang, Ziyan Wu, Chaojun Hu, Fengchun Zhang, Yongzhe Li

**Affiliations:** 1 Department of Rheumatology and Clinical Immunology, Peking Union Medical College Hospital, Chinese Academy of Medical Sciences & Peking Union Medical College, Key Laboratory of Rheumatology and Clinical Immunology, Ministry of Education, Beijing, China; 2 Department of Clinical Laboratory, Beijing Anzhen Hospital, Capital Medical University, Beijing, China; VU University Medical Center, NETHERLANDS

## Abstract

**Objective:**

The anti-carbamylated protein (CarP) antibody is a novel biomarker that might help in the diagnosis of rheumatoid arthritis (RA). We aim to assess the diagnostic value of anti-CarP antibody for RA.

**Methods:**

We systematically searched PubMed, Embase, the Cochrane Library, Web of Science, and Scopus for studies published by December 15, 2015. Studies in any language that evaluated the utility of the anti-CarP antibody in the diagnosis of RA in which healthy donors or patients without arthritis or arthralgia served as controls were included. Two investigators independently evaluated studies for inclusion, assessed study quality and abstracted data. A bivariate mixed-effects model was used to summarize the diagnostic indexes from 7 eligible studies.

**Results:**

The pooled sensitivity, specificity, and positive and negative likelihood ratios for anti-CarP antibody were 42% (95% CI, 38% to 45%), 96% (95% CI, 95% to 97%), 10.2 (95% CI, 7.5 to 13.9), and 0.61 (95% CI, 0.57 to 0.65), respectively. The summary diagnostic odds ratio was 17 (95% CI, 12 to 24), and the area under summary receiver operator characteristic curve was 80% (95% CI, 77% to 84%).

**Conclusion:**

Anti-CarP antibody has a moderate value in the diagnosis of RA with high specificity but relatively low sensitivity.

## Introduction

Rheumatoid arthritis (RA) is a common systemic autoimmune disease, characterized by persistent synovitis, systemic inflammation, and the presence of autoantibodies, particularly anti–cyclic citrullinated peptide (CCP) antibody and rheumatoid factor (RF). RA affects approximately 1% of the population globally[1], and 0.5–1.0% of the adult population in developed countries [2]. The disorder is more prevalent among women older than 65 years. For the development of RA, 50% of the risk is attributable to genetic factors, and the main environmental risk factor is smoking [2].

Irreversible damage to the joints is observed in RA; however early prevention is possible through early diagnosis and treatment. Currently, the anti-CCP antibody and RF are a part of the 2010 American College of Rheumatology (ACR)/The European League Against Rheumatism (EULAR) classification criteria for RA [3, 4]. Despite the diagnostic contribution of the anti-CCP antibody and RF, approximately one-third of patients with RA remain seronegative [5]. Novel serological biomarkers are strongly needed to further improve the diagnosis of seronegative RA.

Anti-carbamylated protein (CarP) antibody, a novel autoantibody, has been detected in RA patients and predicts the development of the pathogenesis of RA, independent of the anti-CCP antibody [6, 7]. This antibody recognizes proteins post-translationally modified by a process of carbamylation, rather than citrullination [8]. Carbamylation occurs when cyanate binds to primary amino or thiol groups presented in the body in equilibrium with urea [8, 9]. The anti-CarP antibody has been described in RA, especially in anti-CCP-negative patients [10, 11]. However, there are controversies regarding the diagnostic accuracy of the anti-CarP antibody in the literature. In this meta-analysis, published data on the sensitivity, specificity, and likelihood ratios of the anti-CarP antibody were summarized for the diagnosis of RA.

## Methods

### Data sources and searches

Without language restrictions, we searched PubMed, Embase, the Cochrane Library, Web of science, and Scopus for studies published by December 15, 2015 that detected the anti-CarP antibody. Our search combined the following index terms: autoantibody to carbamylated protein, autoantibody to CarP, anti-carbamylated protein antibody, anti-CarP antibody, rheumatoid arthritis, RA. The details of the search strategy are listed in the S1 File. We also searched the reference lists of retrieved studies and review articles for additional studies. Our meta-analysis was performed based on the Preferred Reporting Items for Systematic Reviews and Meta-Analyses (PRISMA) guideline (S2 File).

### Study selection

We included studies (1) evaluating the utility of assaying the anti-CarP antibody for the diagnosis of RA; (2) enrolling healthy donors or patients without arthritis or arthralgia as controls; and (3) published that provided enough data to construct a 2×2 table for the diagnostic accuracy of RA. We used the 1987 ACR criteria [12] or the 2010 ACR/EULAR criteria [13] as the diagnostic references. We excluded (1) studies assessing the diagnostic accuracy of the anti-CarP antibody for future RA; (2) studies without valid data after contacting the authors. Two investigators independently scanned titles and abstracts, followed by a full-text review of potential eligible articles.

### Data extraction and study quality assessment

Two investigators independently extracted data by using a standard form that included essential information on the eligible studies, including case number, type of article, trial design, places from which patient groups came, region where the studies were performed, plate and antibody brands of the ELISA, diagnostic criterion, testing method, the demographic characteristics of the participants, control participants, the cut-off of the testing method, and diagnostic indexes. When information from the identified studies was missing, we contacted the authors via email for detailed information. We assessed the study quality according to the Quality Assessment of Diagnostic Accuracy Studies (QUADAS-2) [14]. Discrepancies were resolved through discussion or via consulting professionals.

### Data analysis

We used a bivariate mixed-effects model to combine estimates of sensitivity, specificity, positive and negative likelihood ratios (LR), as well as diagnostic odds ratio (DOR). If heterogeneity existed (P≤0.05 or I^2^≥50%), the heterogeneity test was performed. We constructed a summary receiver operator characteristic (SROC) curve and calculated the area under the SROC curve (AUC) to evaluate the overall performance of the anti-CarP antibody in patients with RA. A sensitivity analysis was performed to evaluate stability by sequential omission of individual studies. Peter’s test was also examined to explore publication bias. For analyses, we used STATA 12.0 software (Stata Corporation, 93 College Station, TX, USA).

## Results

### Search results and characteristics of studies

**Fig 1:**
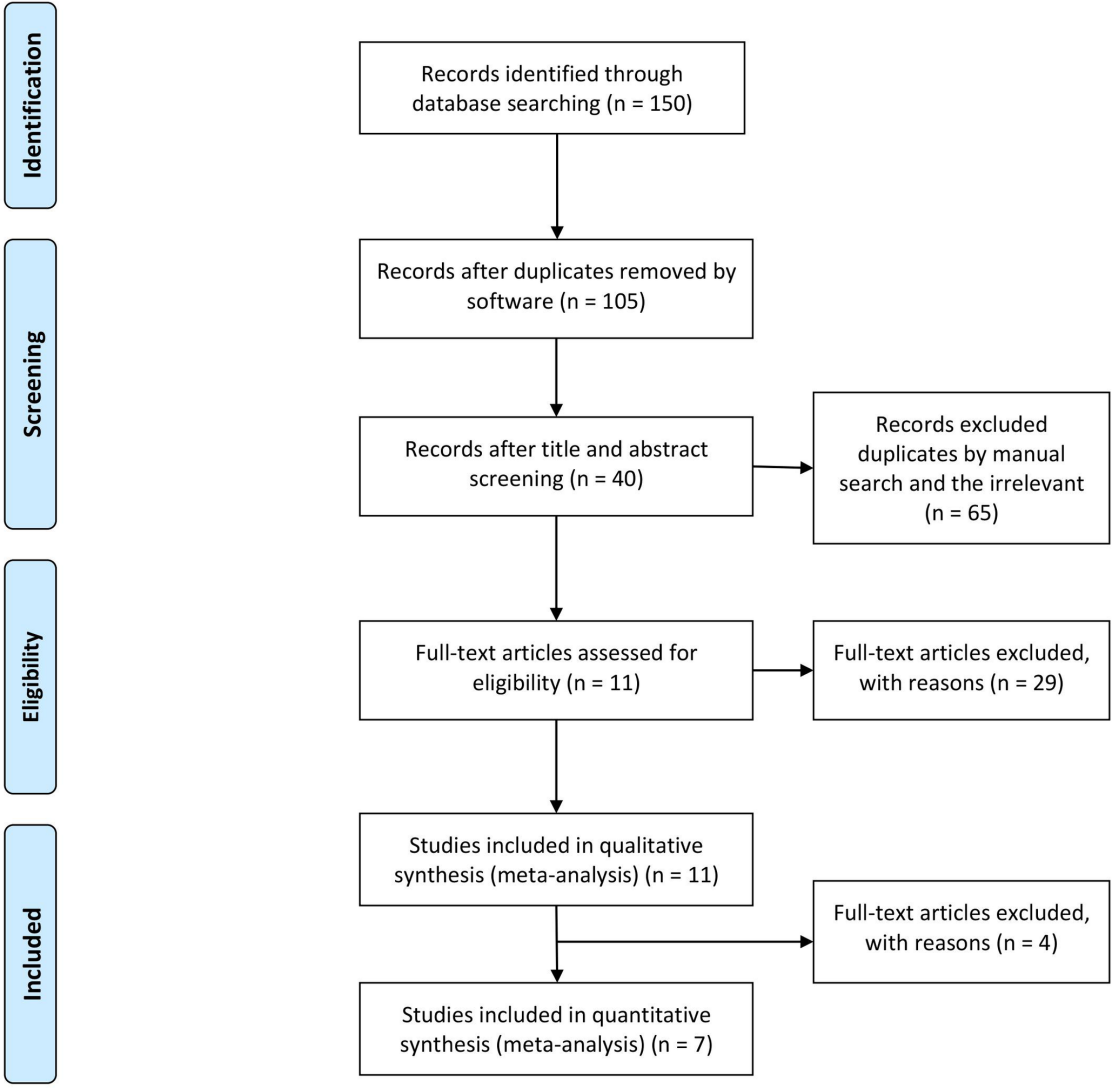
Flow diagram of studies included in the meta-analysis.

We identified 150 published studies, of which 7 studies met the inclusion criteria [11, 15–20]. Fig 1 shows the flow diagram of the study selection process. There were 1898 patients with RA reported on the diagnostic accuracy of the anti-CarP antibody. Table 1 and Table 2 summarize the characteristics of the included studies. Studies were published between 2011 and 2015, including 5 articles, 1 meeting abstract and 1 letter to the editor, as well as 6 documents from Europe and 1 research paper from the USA. We contacted the first author of the meeting abstract [15] and obtained useful information regarding their experiment. After gathering the information from the first author, we assessed the quality of the meeting abstract according to QUADAS-2. The result of the evaluation of the abstract’s quality indicated that the meeting abstract was of high quality, and it was incorporated in the meta-analysis. The letter to the editor also contained enough information to be included. The anti-CarP antibody was identified by ELISA using fetal calf serum (FCS), while binding was determined using IgG. Regarding the characteristics of the control groups, studies used healthy persons as healthy controls and used patients with periodontitis, bronchiectasis, and cystic fibrosis as disease controls. The rationale for the studies included in qualitative synthesis but finally excluded is as follows: (1) three studies [10, 20, 21] did not provide sufficient data to allow the construction of a 2×2 table and (2) one study [22] lacked healthy controls, and only used patients with anti-citrullinated peptide antibody (ACPA)-positive arthralgia and inflammatory arthritis as disease controls. We think that if only patients with arthralgia or arthritis as disease controls were used to construct a 2×2 table for diagnostic accuracy of RA, the results may be biased. Shi et al. [6] reported that of the 340 patients with arthralgia, 133 patients (39%) presented with the anti-CarP antibody. There were 120 (35%) of 340 patients with arthralgia who met the 2010 ACR/EULAR criteria after a median of 12 months. In addition, Shi et al. [23] reported that 26% of the 2086 patients with arthritis were positive for the anti-CarP antibody. A total of 969 (47%) of all of the patients with arthritis met the 2010 ACR/EULAR criteria for RA. Similarly, Humphreys et al. [24] also reported that of 1995 patients with inflammatory polyarthritis, 1221 patients (61%) fulfilled the 2010 ACR/EULAR criteria. The Anti-CarP antibody was present in 460 (23%) of 1995 patients. Therefore, we know that patients with arthralgia or arthritis may subsequently develop RA, and the anti-CarP antibody is detected at a higher rate in these patients. If only patients with arthralgia or arthritis are used as controls, the specificity of the anti-CarP antibody for RA may be inaccurate. Therefore, the study [22] was excluded.

**Table 1:** Some characteristics of the 7 studies included in the meta-analysis of the diagnostic performance of the anti-CarP antibody in rheumatoid arthritis.

Author [Reference]	Year	Case number	Type of article	Design	Patient groups	Region	Plate and antibody brands of ELISA	Criterion	Method
Shi et al. [11]	2011	557	journal article	case-control	Leiden	Netherlands	plate: Thermo Scientific; antibody: DAKO	the 1987 ACR criteria	ELISA
Montes et al. [15]	2014	520	meeting abstract	case-control	Spanish	Spain	NR	the 1987 ACR criteria	ELISA
Challener et al. [16]	2015	212	journal article	case-control	North American	USA	plate: R&D Systems; antibody: Kirkegaard & Perry Laboratories Inc.	the 1987 ACR criteria	ELISA
Brink et al. [17]	2015	192	journal article	case-control	Västerbotten, northern Sweden	Sweden	plate: Nunc; antibody: DAKO	the 1987 ACR criteria	ELISA
Janssen et al. [18]	2015	86	journal article	case-control	Caucasian	Netherlands	plate: Thermo Scientific; antibody: DAKO	established	ELISA
Alessandri et al. [19]	2015	63	journal article	case-control	NR	Italy	plate: Thermo Scientific; antibody: Sigma	the 2010 ACR/EULAR criteria	ELISA
Verheul et al. [20]	2015	268	letter to the editor	case-control	Japanese	Netherlands	plate: Thermo Scientific; antibody: DAKO	the 1987 ACR criteria	ELISA

CarP = carbamylated protein; NR=Not reported; the 1987 ACR criteria=the American Rheumatism Association 1987 revised criteria for the classification of rheumatoid arthritis; the 2010 ACR/EULAR criteria= the 2010 American College of Rheumatology (ACR)/The European League Against Rheumatism (EULAR) classification criteria for RA; ELISA= enzyme-linked immunosorbent assay

**Table 2:** The other characteristics of the 7 studies included in the meta-analysis of the diagnostic performance of the anti-CarP antibody in rheumatoid arthritis.

Author [Reference]	Antigen	Type	RA		Healthy control		Control participants	Cut-off	TP	FP	FN	TN	SEN	SPE
			Female	Median age (yrs)	Female	Median age (yrs)								
Shi et al. [11]	FCS	IgG	NR	NR	NR	NR	Healthy	2SD	250	9	307	296	44.9%	97.0%
Montes et al. [15]	FCS	IgG	76.9%	63	approximately 50%	63	Healthy	2SD	188	10	332	198	36.2%	95.2%
Challener et al. [16]	FCS	IgG	70.3%	Approximately 57	NR	NR	Healthy	2SD	81	3	131	62	38.2%	95.4%
Brink et al. [17]	FCS	IgG	75%	57.5	73.1%	57.5	healthy	a specificity of 97% of ROC curves	81	7	111	190	42.2%	96.4%
Janssen et al. [18]	FCS	IgG	56%	57	60%	26	healthy, periodontitis, bronchiectasis, cystic fibrosis	2SD	41	10	45	261	47.7%	96.3%
Alessandri et al. [19]	FCS	IgG	41%	57.1	NHS: 45% HFDRs: 42%	NHS: 44.6 HFDRs: 54.6	healthy, healthy first-degree relatives of RA patients	3SD	29	14	34	183	46.0%	92.9%
Verheul et al. [20]	FCS	IgG	NR	NR	NR	NR	healthy	a specificity of 97%	121	10	147	314	45.1%	96.9%

CarP = carbamylated protein; NR=Not reported; NHS= normal healthy subjects; HFDRs= healthy first-degree relatives; FCS=fetal calf serum; RA = rheumatoid arthritis; 2SD/3SD= the cut-off for a positive response as the mean plus two/three times the SD of the specific anti-CarP reactivity of the healthy control cohort; ROC= receiver operating characteristic; TP= true positive; FP= false positive; FN= false negative; TN= true negative; SEN=sensitivity; SPE=specificity

### Study quality

**Fig 2:**
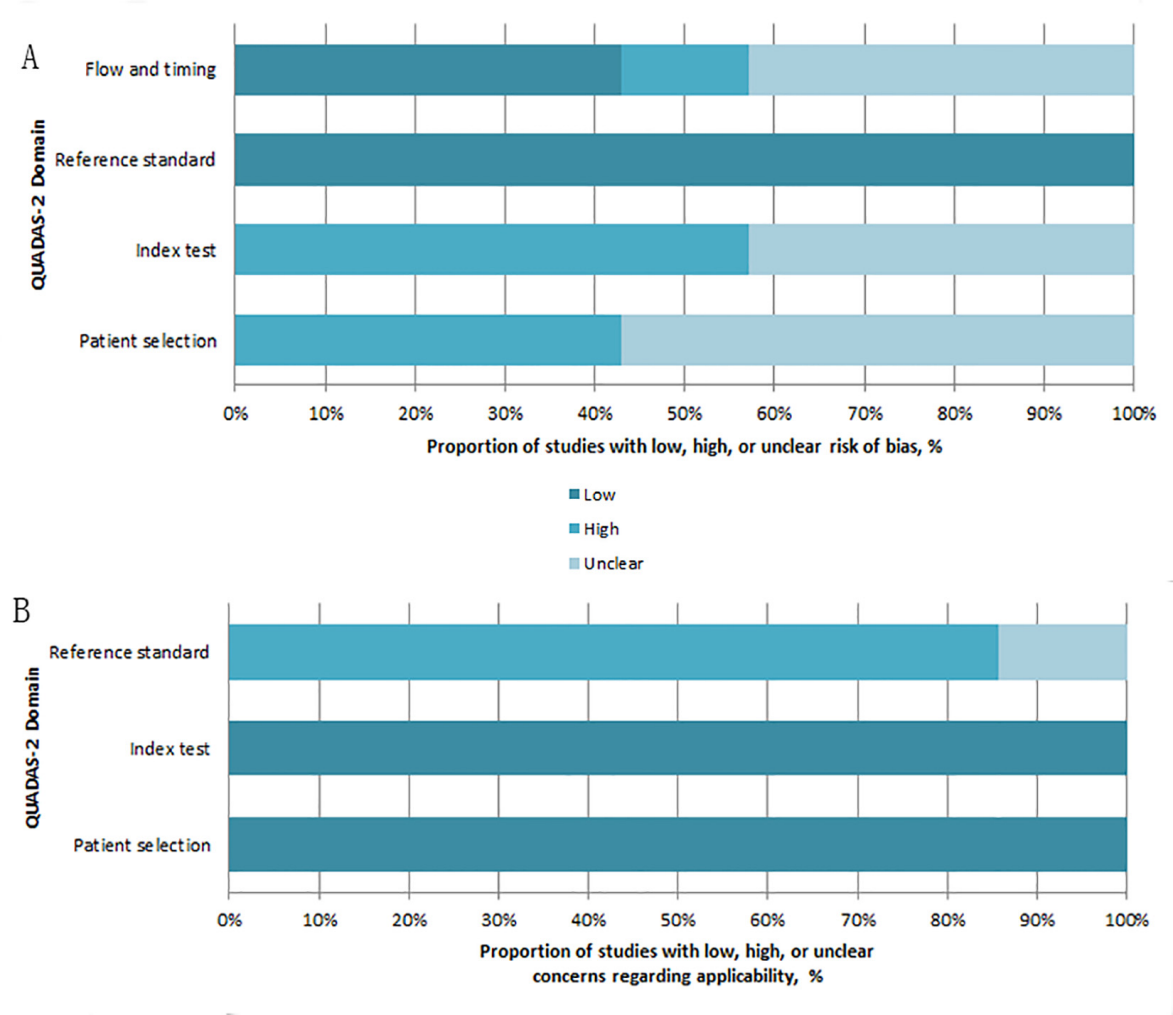
Methodological evaluation according to QUADAS-2 of the included studies.

The graphical display of the evaluation of the risk of bias and concerns regarding applicability of the selected studies, according to QUADAS-2, are reported in Fig 2. Concerning the domain of selection bias, three studies [15, 16, 18] did not explicitly report whether a consecutive or random sample of patients was enrolled. One study [11] did not explicitly report whether inappropriate exclusions were avoided. Four studies [11, 15–17] showed that the conduct or interpretation of the index test could introduce high bias, and three studies [18–20] introduced unclear bias. Regarding the domain of flow and timing, one study [15] did not explicitly report whether there was an appropriate interval between the index test and reference standard, while another study [18] did not explicitly indicate the reference standard used. One study [11] did not explicitly report whether all patients were included in the analyses. There were minimal concerns about patient selection and index test applicability, but unclear concern about the applicability of the reference standard in one study [18] as well as high concern in the remaining six studies. Overall, none of the 7 included studies indicated large methodological flaws, which would warrant their exclusion from the meta-analysis.

### Diagnostic accuracy of anti-CarP antibody

**Fig 3:**
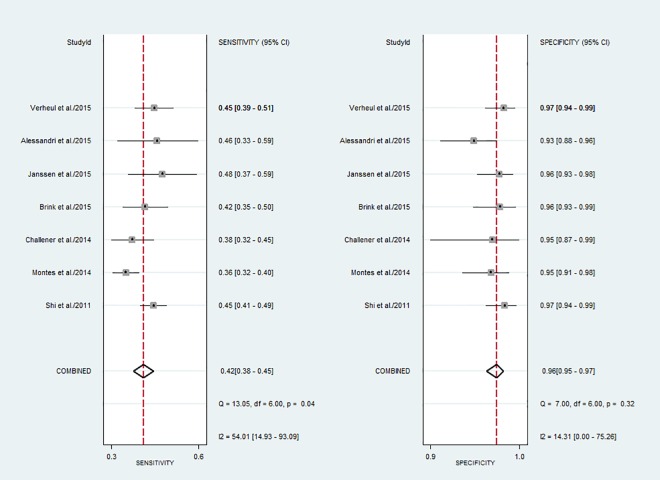
Forest plot of the sensitivity and specificity of the anti-CarP antibody in the diagnosis of rheumatoid arthritis.

**Fig 4:**
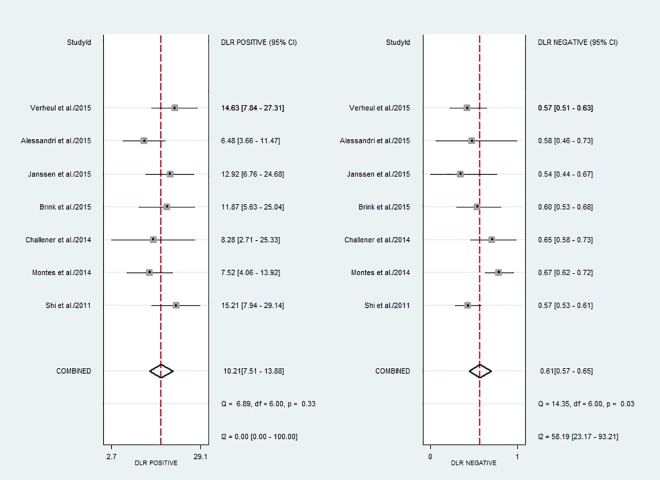
Forest plot of the positive and negative likelihood ratios of the anti-CarP antibody in the diagnosis of rheumatoid arthritis.

**Fig 5:**
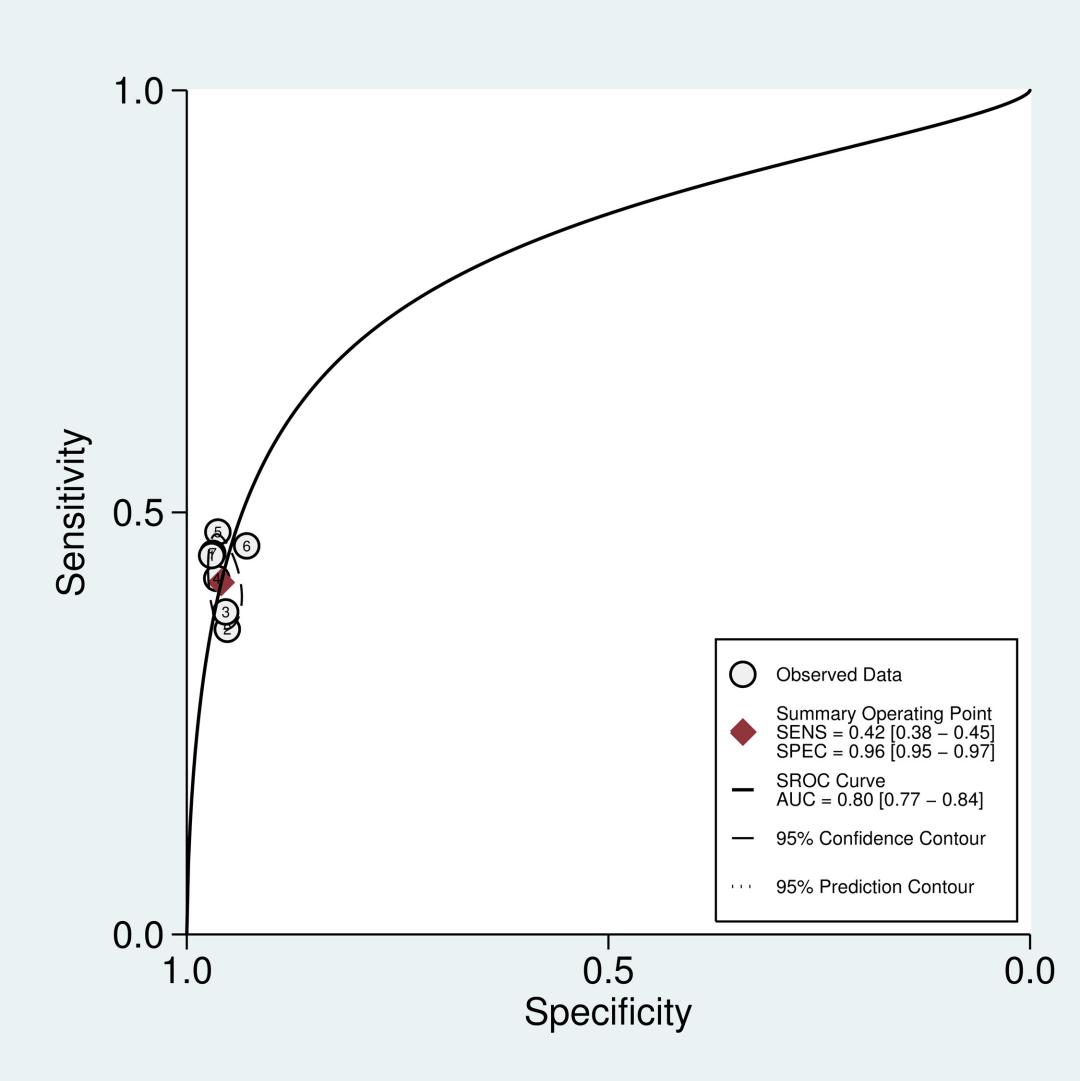
SROC of the accuracy of the anti-CarP antibody in the diagnosis of rheumatoid arthritis.

The diagnostic sensitivity of the anti-CarP antibody testing ranged from 36.2% to 47.7%, while the specificity ranged from 92.9% to 97.0%. The pooled sensitivity and specificity were 42% (95% CI, 38% to 45%) and 96% (95% CI, 95% to 97%), respectively. Fig 3 shows the forest plot of the diagnostic sensitivity and specificity of the anti-CarP antibody in the diagnosis of RA from included studies. The summary positive LR and summary negative LR, respectively, were 10.2 (95% CI, 7.5 to 13.9) and 0.61 (95% CI, 0.57 to 0.65) (Fig 4). The summary DOR was 17 (95% CI, 12 to 24), and the AUC was 80% (95% CI, 77% to 84%) (Fig 5).

### Exploration of heterogeneity and publication bias

**Fig 6:**
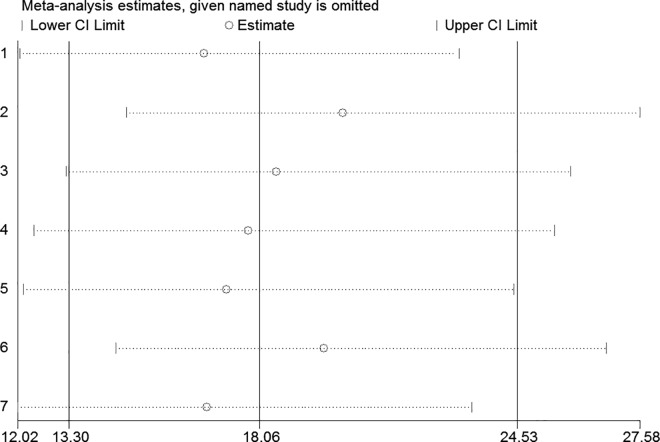
Sensitivity analysis of the included studies.

We used a bivariate mixed-effects model in this meta-analysis (P = 0.471, I^2^ = 0). No significant heterogeneity was found among the included studies. A sensitivity analysis indicated that the results of this meta-analysis were stable (Fig 6). Conducting Peter’s test did not reveal any small-study effects or publication bias in this study (P = 0.437).

## Discussion

RA can cause functional disability and reduce the quality of life. Early and accurate treatment interventions play an important role in preventing the development of massive erosions and deformities in the inflamed joints of RA patients. Specific laboratory tests are desirable to help in the early identification of RA. Robust biomarkers are also recommended for early diagnosis of RA to increase the benefits from aggressive interventions. The anti-CarP antibody was one of the newly discovered autoantibodies in the sera of patients with RA. Research by Shi J et al. shows that anti-CarP and anti-CCP antibodies are different, but some cross-reactivity may exist [25]. Anti-CarP-FCS may have less cross-reactivity to citrullinated proteins than anti-carbamylated-fibrinogen (Fib) [26]. Thus far, several studies have reported that the anti-CarP antibody may provide additional benefit in the diagnosis of RA, especially for those with early and anti-CCP-negative diseases [5, 9–11, 27, 28]. The anti-CarP antibody are also detected juvenile idiopathic arthritis [10, 22, 29], psoriatic arthritis [30], and systemic sclerosis [31]. The association between the anti-CarP antibody and radiographic progression is strong in the total RA population as well as in the ACPA-negative subgroup [32]. The anti-CarP antibody may be used to predict radiographic progression within the RA population. Thus, the detection of the anti-CarP antibody may be a useful serological test for the identification and sub-classification of patients with RA.

This meta-analysis is the first study to evaluate the diagnostic value of the anti-CarP antibody for RA. We included 7 studies in this meta-analysis. The potential diagnostic accuracy of the anti-CarP antibody was mainly due to its high specificity (96%) and positive LR (10.2), suggesting that patients with RA had a 10.2-fold higher chance of having a positive anti-CarP antibody test compared to controls. The sensitivity was lower (42%), while the negative LR was not low enough (negative LR<0.1) to exclude RA when the anti-CarP antibody tests were negative. The DOR (17) indicated that serum levels of the anti-CarP antibody could be helpful in diagnosing RA. Similarly, the AUC (80%) demonstrated that the anti-CarP antibody had a moderate diagnostic value for RA. Furthermore, 5 larger sample studies (cases≥100) [11, 15–17, 20] were separately analyzed to evaluate the diagnostic value of anti-CarP antibody for RA. The pooled sensitivity, specificity, positive LR and negative LR were 41% (95% CI, 38% to 45%), 96% (95% CI, 95% to 97%), 11.4 (95% CI, 7.8 to 16.5) and 0.61 (95% CI, 0.57 to 0.65), respectively. The summary DOR was 19 (95% CI, 12 to 28), and the AUC was 84% (95% CI, 81% to 87%). The results of the larger sample studies are identical to the overall results.

In clinical applications, there are limitations of the 2010 ACR/EULAR classification criteria of RA with high sensitivity, such as failing to capture cases of symmetrical seronegative arthritis and limited joint involvement [11, 20, 33]. Anti-carbamylated-FCS IgG and IgA antibodies are present in both anti-CCP negative (IgG: 16%, IgA: 30%) and anti-CCP positive (IgG: 73%, IgA: 51%) RA patients [9]. The first presentation of the anti-CarP antibody was comparable with the anti-CCP antibody and presented earlier than IgM RF [27]. Therefore, the anti-CarP antibody might be a potential biomarker to identify anti-CCP-negative patients, who may benefit from early and aggressive interventions [34]. In addition, the simultaneous assessment of anti-CarP, anti-CCP, and RF might be beneficial in identifying RA.

Furthermore, other novel biomarkers found in patients with RA have been studied for their diagnostic value of RA. We summarized diagnostic performance of various antibody assays of RA in Table 3.

**Table 3:** Diagnostic performance of various antibodies assays in rheumatoid arthritis.

[Reference]	Antigen	SEN (%)	SPE (%)	Positive LR	Negative LR	Supplementary information
[35] [36] [37]	CCP	30 to 70	91 to 99	12.46	0.36	—
[35, 38]	IgM RF	69	85	4.86	0.38	RF occurs in 60 to 80% of established and 50 to 60% of early RA
[35]	CCP1	—	—	13.03	0.53	—
[35] [39–41]	CCP2	—	—	12.77	0.32	Positive in 20 to 30% RF-negative RA patients
[35]	IgA RF	—	—	5.01	0.44	—
[35]	IgG RF	—	—	4.52	0.52	—
[39] [40] [42, 43]	MCV	60 to 77	87 to 98	7.24	0.28	occur in 21–43% of RA patients
[5]	Savoie	40	92 to 98	—	—	positive in approximately 43% of RA patients; positive in 27% RF-negative RA patients
[25]	IgA CarP	43	95	—	—	—

SEN = sensitivity; SPE = specificity; LR = likelihood ratios; CCP = cyclic citrullinated peptide; RF = rheumatoid factor; RA = rheumatoid arthritis; MCV = mutated citrullinated Vimentin; CarP = carbamylated protein.

We excluded other RA-associated autoantibodies that had high specificity, such as anti-perinuclear factor and antikeratin antibodies [44, 45], because rigorous technical requirements are needed for their detection. Compared with the antibodies in Table 3, the specificity of the anti-CarP antibody was high; however, the sensitivity was lower than that of the anti-CCP antibody and RF. It is possible that the assays used to detect these antibodies may differ in definition based on cut-off and diagnosis indexes, which may potentially limit their comparability. However, because the anti-CCP antibody has a high clinical utility, it is unlikely that the anti-CCP antibody will be replaced by new markers discovered in recent years.

Our position on the anti-CarP antibody is listed as follows: (1) The anti-CarP antibody is a novel biomarker that was recently discovered and, needs further studies to define its clinical utility; (2) The anti-CarP antibody is present before symptoms develop [5, 46, 47], suggesting that more research is necessary to determine its prognostic value; (3) The role of anti-CarP antibody in the immunological mechanisms of RA pathogenesis needs further study. So far, there has been research on this issue [48–51]; (4) According to the 2010 ACR/EULAR criteria, the weights for anti-CCP antibody and RF varied due to the titers. In addition, it needs to be determined whether a relationship exists between anti-CarP antibody titers and its diagnostic accuracy; and (5) Thus far, there has been no report in the literature of analysis of serum level of anti-CarP antibody between RA patients and relevant disease patients, who would be seen in rheumatology clinics. Our meta-analysis focused on healthy donors as controls. It is necessary to conduct research on the positive rate of the anti-CarP antibody in sera of patients with different diseases, which will allow a greater understanding of the diagnostic value of this antibody.

Our meta-analysis has some limitations. First, some articles published in other databases may have been missed. Second, certain concerns were raised during our assessment of the quality of the studies. For example, some included studies did not report whether the detection results of the anti-CarP antibody were performed blindly, which may cause measurement bias. However, these concerns did not influence our results as we found no heterogeneity or publication bias. The current studies were case-control studies. Well-designed prospective studies with larger sample sizes are needed to further confirm the value of the anti-CarP antibody for RA. In addition, in the present studies, the population was predominantly Caucasian, while only one study involved an Asian population. There were no Africans in the populations studied.

In conclusion, the anti-CarP antibody has a moderate diagnostic value, with high specificity but relatively low sensitivity in the diagnosis of RA.

## Supporting Information

S1 File The search strategy using the five databases. (DOCX)

S2 File PRISMA checklist. (DOC)
