# Supplementary material for: Meta-Analysis: Diagnostic Accuracy of Anti-Carbamylated Protein Antibody for Rheumatoid Arthritis
Source: PLoS One. 2016 Jul 20;11(7):e0159000. doi: 10.1371/journal.pone.0159000 (PMC4954701; doi:10.1371/journal.pone.0159000)
Supplement: S1 File — (DOCX) [file pone.0159000.s001.docx]

**S1 The search strategy using the five databases**

1. The search strategy in Pubmed

(((((((((autoantibody to carbamylated protein[Title/Abstract]) OR auto-antibody to carbamylated protein[Title/Abstract]) OR autoantibody to CarP[Title/Abstract]) OR auto-antibody to CarP[Title/Abstract]) OR anti carbamylated protein antibody[Title/Abstract]) OR anti-carbamylated protein antibody[Title/Abstract]) OR anti CarP antibody[Title/Abstract]) OR anti-CarP antibody[Title/Abstract])) AND ((((rheumatoid arthritis[Title/Abstract]) OR RA[Title/Abstract])) OR "Arthritis, Rheumatoid"[Mesh])

1. The search strategy in Embase

| No. | Query | Results | Date |
| --- | --- | --- | --- |
| #14 | 'autoantibody'/exp OR autoantibody AND to AND carbamylated AND ('protein'/exp OR protein) OR ('auto antibody'/exp OR 'auto antibody' AND to AND carbamylated AND ('protein'/exp OR protein)) OR ('autoantibody'/exp OR autoantibody AND to AND ('carp'/exp OR carp)) OR ('auto antibody'/exp OR 'auto antibody' AND to AND ('carp'/exp OR carp)) OR (anti AND carbamylated AND ('protein'/exp OR protein) AND ('antibody'/exp OR antibody)) OR ('anti carbamylated' AND ('protein'/exp OR protein) AND ('antibody'/exp OR antibody)) OR (anti AND ('carp'/exp OR carp) AND ('antibody'/exp OR antibody)) OR ('anti carp' AND ('antibody'/exp OR antibody)) AND ('rheumatoid arthritis'/exp OR (rheumatoid AND ('arthritis'/exp OR arthritis)) OR ra) | 62 | 15-Dec-15 |
| #13 | 'rheumatoid arthritis'/exp OR (rheumatoid AND ('arthritis'/exp OR arthritis)) OR ra | 233013 | 15-Dec-15 |
| #12 | 'autoantibody'/exp OR autoantibody AND to AND carbamylated AND ('protein'/exp OR protein) OR ('auto antibody'/exp OR 'auto antibody' AND to AND carbamylated AND ('protein'/exp OR protein)) OR ('autoantibody'/exp OR autoantibody AND to AND ('carp'/exp OR carp)) OR ('auto antibody'/exp OR 'auto antibody' AND to AND ('carp'/exp OR carp)) OR (anti AND carbamylated AND ('protein'/exp OR protein) AND ('antibody'/exp OR antibody)) OR ('anti carbamylated' AND ('protein'/exp OR protein) AND ('antibody'/exp OR antibody)) OR (anti AND ('carp'/exp OR carp) AND ('antibody'/exp OR antibody)) OR ('anti carp' AND ('antibody'/exp OR antibody)) | 263 | 15-Dec-15 |
| #11 | ra | 101436 | 15-Dec-15 |
| #10 | rheumatoid AND ('arthritis'/exp OR arthritis) | 181613 | 15-Dec-15 |
| #9 | 'anti carp' AND ('antibody'/exp OR antibody) | 79 | 15-Dec-15 |
| #8 | anti AND ('carp'/exp OR carp) AND ('antibody'/exp OR antibody) | 240 | 15-Dec-15 |
| #7 | 'anti carbamylated' AND ('protein'/exp OR protein) AND ('antibody'/exp OR antibody) | 34 | 15-Dec-15 |
| #6 | anti AND carbamylated AND ('protein'/exp OR protein) AND ('antibody'/exp OR antibody) | 63 | 15-Dec-15 |
| #5 | 'auto antibody'/exp OR 'auto antibody' AND to AND ('carp'/exp OR carp) | 66 | 15-Dec-15 |
| #4 | 'autoantibody'/exp OR autoantibody AND to AND ('carp'/exp OR carp) | 67 | 15-Dec-15 |
| #3 | 'auto antibody'/exp OR 'auto antibody' AND to AND carbamylated AND ('protein'/exp OR protein) | 43 | 15-Dec-15 |
| #2 | 'autoantibody'/exp OR autoantibody AND to AND carbamylated AND ('protein'/exp OR protein) | 44 | 15-Dec-15 |
| #1 | 'rheumatoid arthritis'/exp | 165802 | 15-Dec-15 |

1. The search strategy in the Cochrane Library

| ID | Search | Hits |
| --- | --- | --- |
| #1 | MeSH descriptor: [Arthritis, Rheumatoid] explode all trees | 4207 |
| #2 | autoantibody to carbamylated protein:ti,ab,kw (Word variations have been searched) | 0 |
| #3 | auto-antibody to carbamylated protein:ti,ab,kw (Word variations have been searched) | 0 |
| #4 | autoantibody to CarP:ti,ab,kw (Word variations have been searched) | 0 |
| #5 | auto-antibody to CarP:ti,ab,kw (Word variations have been searched) | 0 |
| #6 | anti carbamylated protein antibody:ti,ab,kw (Word variations have been searched) | 0 |
| #7 | anti-carbamylated protein antibody:ti,ab,kw (Word variations have been searched) | 0 |
| #8 | anti CarP antibody:ti,ab,kw (Word variations have been searched) | 0 |
| #9 | anti-CarP antibody:ti,ab,kw (Word variations have been searched) | 0 |
| #10 | #2 or #3 or #4 or #5 or #6 or #7 or #8 or #9 | 0 |
| #11 | rheumatoid arthritis:ti,ab,kw (Word variations have been searched) | 7644 |
| #12 | RA:ti,ab,kw (Word variations have been searched) | 4361 |
| #13 | #1 or #11 or #12 | 9092 |
| #14 | #10 and #13 | 0 |

1. The search strategy in Web of Science

TS=(autoantibody to carbamylated protein OR auto-antibody to carbamylated protein OR autoantibody to CarP OR auto-antibody to CarP OR anti carbamylated protein antibody OR anti-carbamylated protein antibody OR anti CarP antibody OR anti-CarP antibody) AND TS=(rheumatoid arthritis OR RA)

1. The search strategy in Scopus

( TITLE-ABS-KEY ( autoantibody to carbamylated protein OR auto-antibody to carbamylated protein OR autoantibody to carp OR auto-antibody to carp OR anti carbamylated protein antibody OR anti-carbamylated protein antibody OR anti carp antibody OR anti-carp antibody ) AND TITLE-ABS-KEY ( rheumatoid arthritis OR ra ) )
